# Baby Bump or Baby Slump? COVID-19, Lockdowns, and their Effects on Births in Australia

**DOI:** 10.1016/j.ssmph.2024.101604

**Published:** 2024-01-05

**Authors:** Irma Mooi-Reci, Mark Wooden, Federico Zilio

**Affiliations:** a School of Social and Political Sciences, Level 5, John Medley Bldg, The University of Melbourne, Victoria, 3010, Australia; b Melbourne Institute: Applied Economic & Social Research, University of Melbourne, Victoria, 3010, Australia

**Keywords:** Australia, Births, COVID-19, Lockdowns

## Abstract

This study examines changes in birth rates in Australia during the COVID-19 pandemic and the extent to which such changes were influenced by lockdowns. We use natality data at State and small regional area levels spanning the period from 2011 to 2022. In our empirical approach, we first take advantage of a unique quasi-experimental setting that arose in Victoria, Australia's second most populous State, during the first year of the pandemic. Victoria imposed a 111-day stay-at-home lockdown while other States and Territories enforced milder restrictions on social and economic activities. We then exploit lockdowns that lasted more than three months in Victoria and New South Wales in the second year of the pandemic. Within these quasi-experimental settings, our empirical approach was to first use monthly data at the State-level and estimate birth rate deviations from secular trends for the months affected by COVID-19 policies. We also estimate separate models to examine variations in births across regional areas with different compositions of Indigenous population, unemployment, low-income, and non-English speaking residents. Our findings reveal a nationwide fertility increase in 2021, but Victoria exhibited slower growth, especially in areas with higher unemployment, lower income, and more non-English speaking residents. In 2022, we find evidence of a gradual return of birth rates to pre-pandemic trends, though this is mainly concentrated in the major cities. While the second-year lockdowns had limited impacts, language-diverse areas still mostly experienced lower rates of growth in birth rates.

## Highlights

• Various studies predicted that fertility would drop following the COVID-19 pandemic.
• However, evidence predicting whether these predictions indeed translated to drops in real birth rate, remains mixed.
• Australia experienced a temporary baby bump with birth rates gradually returning to pre-pandemic trends.
• However, birth rates in Victoria and New South Wales, the States that imposed stricter lockdowns, grew at a slower pace.
• Slower growth in birth rates were associated with high unemployment, high non-English speaking and low income areas.

## 1 Introduction

Findings from survey-based studies mostly suggest that, following the outbreak of the COVID-19 pandemic, many fertile people put plans to have a child or another child on hold (e.g., Emery & Koops, 2022; Kahn et al., 2021; Lazzari et al., 2023; Lin et al., 2021; Luppi et al., 2020; Mooi-Reci et al., 2023). However, evidence about how this decline in reported fertility intentions has translated into a decline in actual births remains mixed.

The earliest studies of the association between the COVID-19 pandemic and birth rates (e.g., Aassve, Cavalli, Mencarini, Plach, & Sanders, 2021) highlighted negative associations. Subsequent research, however, reported that such negative effects were mostly confined to a few months at the end of 2020 and the beginning of 2021 (e.g., Bailey et al., 2023; Kearney & Levine, 2022; Pomar et al., 2022). In the US, Bailey et al. (2023) reported that much of the initial decline in fertility was driven by a sharp reduction in births to foreign-born mothers and that among US-born mothers the fertility rate in 2021 was 5.1% higher than its pre-pandemic trend, with fertility improvements especially pronounced among university-educated women. Very differently, Sobotka et al. (2023) analyzed fertility trends of 38 higher-income countries and showed that fertility patterns have varied substantially across countries. After an initial decline, fertility improved in 2021 in the US, Canada, the UK, New Zealand, and North-European countries, while most other countries experienced a decline in fertility.^1^ However, many countries experienced another decline in births in 2022, potentially indicating ongoing challenges influencing birth rates during the later stages of the pandemic. In Australia, aggregate data for 2021 reveal trends that are broadly in line with the European and North American experience (Gray et al., 2022), but it is unclear whether these trends persisted beyond 2021.

This study adds to this body of research by using monthly and annual data on registered births in Australia to estimate simple statistical models that quantify how much birth rates changed over the period affected by COVID-19. Our analysis focuses on women aged 15 to 45 and spans the period 2011 to 2022. We add to Gray et al. (2022) by quantifying the extent to which birth rates over the period December 2020 to June 2022 (so roughly more than 9 months after the introduction of COVID-19 restrictions) deviates from pre-pandemic trends. Additionally, we split the pandemic into two periods, before and after the commencement of the COVID-19 vaccination program, which in Australia began in February 2021. Since the stringency of containment measures varied across different Australian States and Territories, with residents in some States (Victoria in 2020 and New South Wales and Victoria in 2021) subject to far more days in lockdowns than residents of any other State, we exploit cross-State differences to shed light on the role played by lockdowns in affecting birth rates.

This focus on birth rates sets our study apart from other recent Australian studies on the impact of the COVID-19 pandemic (Lazzari et al., 2023; Mooi-Reci et al., 2023), where the outcome has been fertility intentions. We analyze birth rates at the aggregate level, which helps in understanding broad demographic shifts that are otherwise obscured when examining experiences and decision-making processes at the individual level. We also examine whether the differences in birth rates from pre-pandemic trends vary with selected socio-economic characteristics of the areas in which people live. More specifically, we test for heterogeneity with respect to location (metropolitan vs regional), unemployment rates, median household income, the proportion of Indigenous persons, and the proportion of non-English speakers.

### 1.1 COVID-19, lockdowns, and birth outcomes: Theoretical expectations

The decision to have a child is influenced by various factors. Among these factors, the presence of a healthy and safe environment, along with stable socioeconomic conditions, play a crucial role. While the factors influencing fertility are manifold (Voicu & Bădoi, 2021), it remains unclear a priori whether the COVID-19 pandemic and the accompanying lockdowns led to higher or lower birth rates.

On one hand, it can be argued that the COVID-19 pandemic immediately disrupted the safe and healthy environment necessary to start or expand a family. Particularly during the first year of the pandemic, concerns about the spread of the virus and the potential impact on the well-being of both parents and their future children prompted some couples to delay their plans to conceive children (e.g., Emery & Koops, 2022; Kahn et al., 2021; Luppi et al., 2020; Mooi-Reci et al., 2023). Furthermore, increased pressure on healthcare systems likely meant that many couples requiring reproductive health services could not be assisted (Bailey et al., 2023), potentially preventing them from having children. The first months of the pandemic were also associated with policy responses that led to a high number of business closures and a surge in job losses (Bartik et al., 2020). Previous experiences from economic downturns and public health crises have shown that pregnancies and birth rates tend to decline in response to economic uncertainty (Autor et al., 2019; Schaller, 2016). Typically, concerns about job prospects generate a feeling of uncertainty about the future, prompting individuals and couples to either defer or postpone starting a family (see Balbo et al., 2013).

Aside from health and economic factors, social distancing measures and lockdowns to contain the spreading of the virus disrupted individuals’ intimate relationships (e.g., Lehmiller et al., 2021). Specifically, uncertainty about the length of the lockdowns increased stress and anxiety (e.g., Brodeur et al., 2021) and had negative effects on mental health (e.g., Butterworth et al., 2022). These factors, in turn, may have negatively impacted family plans and influenced patterns of sexual interactions and contraceptive use (Lehmiller et al., 2021).

Lockdowns also had indirect effects on fertility behavior due to the closure of schools, placing additional strain on families to manage childcare responsibilities. As schools and childcare centers closed, women with children were pushed to increase the share of their domestic work and care for their children due to limited family support and home-schooling demands (Carlson, Petts, & Pepin, 2022; Craig & Churchill, 2021; Sevilla & Smith, 2020). This unequal distribution of paid and unpaid work reshaped work and family responsibilities during lockdowns and potentially contributed to a decrease in new births, particularly among parents of young children.

Research also suggests that the burden of the lockdowns was not distributed evenly across socioeconomic and demographic groups (Adams-Prassl et al., 2020; Perry et al., 2021). Job losses and economic hardship disproportionately affected women, the less educated and those in lower income strata who were employed in industries that were hardest hit by the pandemic (ILO, 2020). COVID-related health risks were greater among people of color, while older workers and those already out of paid employment were confronted with uncertain economic futures making it difficult to face decisions about having children. We thus hypothesize that areas with high unemployment and low income, as well as areas with a higher proportion of migrants and Indigenous persons, will be associated with larger declines in birth rates during the pandemic than other areas.

However, counterbalancing these adverse factors were mitigating circumstances that might have slowed the rate of decline in fertility. In particular, in most advanced economies the pandemic was accompanied by unprecedented government income support measures. This was especially the case in Australia, where the federal government massively (but temporarily) expanded the level of income support it provided to the working-age population. In addition, the COVID-19 pandemic and ensuing lockdowns led to a significant shift in work arrangements, with a notable increase in the number of people working from home. Data from the HILDA Survey reveals that 24% of Australians worked from home most of the time during the pandemic compared with only about 6% pre-pandemic. We argue that the associated reduction in commuting time likely increased the amount of time couples spent together, potentially leading to enhanced relationship satisfaction and subsequently an increase in actual birth rates. It has also been suggested that restrictions on mobility, especially in the early stage of the pandemic, led to disruptions in access to contraception and abortion (Sobotka et al., 2023), which in turn could contribute to higher fertility.

Overall, whether the COVID-19 pandemic and accompanying lockdowns resulted in higher or lower birth rates in Australia is an empirical question, and one this study seeks to answer.

### 1.2 The Australian context

Like other countries, Australian governments responded to the outbreak of the COVID-19 pandemic by announcing a wide range of sweeping measures intended to contain the spread of the virus (see Stobart & Duckett, 2022). But very different to most other high-income countries, these policies proved very effective at minimizing the risk of infection, with less than 30,000 COVID-19 cases and fewer than 1000 deaths recorded during the first year of the pandemic. In large part, this reflects both the introduction of strict border controls, with arrivals into the country largely restricted to returning Australians (who were then required to enter an official quarantine facility or hotel upon arrival), and the relative ease with such border restrictions can be enforced in Australia. Australian governments also introduced measures designed to restrict mobility within Australia. A nationwide partial lockdown commenced in late March 2020, which involved, among other things, the closure of many non-essential businesses, a move to online learning within schools and universities, and advice to work from home where possible. By early May, this lockdown had come to an end, but many other measures (e.g., mask-wearing mandates in certain settings, capacity limits on public venues, and restrictions on inter-State travel) remained in place. Importantly, during the second half of 2020, persons living in Australia's second most populous State, Victoria (accounting for around one-quarter of Australia's population), were subject to another, and far more stringent, lockdown, that for residents of its main city, Melbourne, would last for 111 days. In contrast, residents in other States were for the most part free of lockdowns. After exiting lockdown in late October 2020, Victoria experienced similar looser restrictions to those in place in other States. The Australian federal government began the rollout of the COVID-19 vaccines on February 21, 2021. An outbreak of the Delta variant forced the Australian most populous State, New South Wales, to declare a state of lockdown until October 11, 2021. A similar outbreak soon followed in Victoria, leading to the imposition of yet further lockdowns that were not lifted until the State reached the threshold of 70% of the adult population double-dose vaccinated (in late October). The unvaccinated remained under stay-at-home orders until December 15.

Additionally, and as noted above, Australian governments significantly increased public spending on programs designed to provide financial relief to the working-age population. Most important here were an $88 billion wage subsidy program (known as JobKeeper), early access to private pension (superannuation) accounts (which resulted in an additional $38 billion for households), and $52 billion in additional income payments supporting the unemployed, students and parents (Breunig & Sainsbury, 2023). Indeed, Breunig and Sainsbury (2023) estimate that 6.5 million Australians (42% of the working age population) received some form of payment, and the median recipient had 46% of their pre-COVID-19 wages replaced by income support.

## 2 Materials and methods

### 2.1 Data

We use data on birth registrations and population estimates for the years 2011–2022 published by the Australian Bureau of Statistics (ABS) (ABS 2023a; 2023c). The births data include records from both monthly State/Territory birth counts and annual birth counts by small regional area.^2^ Monthly State/Territory birth counts refer to the date of birth of the newborn whereas annual birth counts are based on the date of birth registration. Because of delays in birth registration, there will, however, be discrepancies between these two measures. More specifically, almost 12% of the births registered in 2021 referred to births that did not occur in 2021. For 2022, almost 15% of the registered births occurred in 2021 or earlier. To address this issue, we limit the analysis of date of birth monthly counts to data up to June 2022, thus excluding births between July and December 2022 that are most at risk of being registered in 2023. Annual birth counts by small regional areas are used to estimate the differential impact of COVID-19 measures among communities with diverse socioeconomic and demographic characteristics. Counts of females aged 15 to 45 are calculated from ABS population estimates available by gender and age (ABS 2023c). The population estimates for States and Territory are provided quarterly and those for SA2 areas on an annual basis.

### 2.2 Methods

Our measure of fertility is the birth rate, defined as the number of births per thousand females aged 15 to 45. When we use monthly date of birth data by State/Territory, this is calculated as:

$${BR}_{smy}=\frac{B_{smy}*1,000}{{FP}_{smy}}$$

where $B_{smy}$ and ${FP}_{smy}$ are, respectively birth counts, and the estimated female population aged 15 to 45 in State/Territory *s* in month *m* of year *y*. The estimate of the monthly female population is calculated from quarterly population data as follows:

• For the first month of a quarter, it is two-thirds of the population of the quarter and one-third of the population of the preceding quarter.
• For the second month, it corresponds to the population of the quarter.
• For the third month of a quarter, it is two-thirds of the population of the quarter and one-third of the population of the subsequent quarter.

Birth rates by SA2 area ($B_{asy}$) are annual rates calculated as:

$${BR}_{asy}=\frac{B_{asy}*1,000}{{FP}_{asy}}$$

where $B_{asy}$ is birth counts and ${FP}_{asy}$ is the estimated female population aged 15 to 45 in SA2 area *an* of State/Territory *s* in year *y*. As birth rates of less densely populated areas are sensitive to small changes in birth counts, the analysis omits SA2 areas with fewer than 500 females aged 15 to 45. This results in the exclusion of 168 SA2 areas (6% of all SA2 areas).

Birth rate changes in the period affected by COVID-19 are derived from ordinary least squares (OLS) regressions that estimate the deviations from the State/Territory trends that birth rates would have followed in the absence of the COVID-19 pandemic. For the first phase of the COVID-19 pandemic we assume that birth rates will start deviating from past trends in December 2020, which is a little more than nine months after the Australian government declared a human biosecurity emergency (on March 18, 2020) and began introducing restrictions on economic and social activity intended to slow the spread of the COVID-19 virus. We also test the hypothesis of a differential impact in Victoria, which experienced tougher and far more prolonged restrictions than other States. We set the start of the second phase of the pandemic in December 2021, nine months after the Australian government began the roll-out of the COVID-19 vaccine. For this second period we assess whether the birth rates of the two States that experienced prolonged lockdowns (New South Wales and Victoria) were affected differently than the rates of the States with milder restrictions. In measuring the impacts of COVID-19 measures on birth rates we estimate three specifications.

In specification 1, we use monthly date of birth data to estimate the average change in birth rates over the period between December 2020 and June 2022. The estimated regression is:

$$\log\left({BR}_{smy}\right)=\alpha +\gamma_{s}+\delta_{s}tr+\mu_{m}+\sum_{age=1}^{6}\theta_{age}{\mathrm{Pr}\_F\_age}_{smy}$$

$$+\left(\beta_{A;1}{VIC}_{s}+\beta_{A;2}{NSW}_{s}+\beta_{A;3}{Oth\_St}_{s}\right)*{First\_COVID}_{my}$$

$$+\left(\beta_{B;1}{VIC}_{s}+\beta_{B;2}{NSW}_{s}+\beta_{B;3}{Oth\_St}_{s}\right)*{Second\_COVID}_{my}+\varepsilon_{smy}$$

where ${BR}_{smy}$ is birth rate of State *s* in month m of year *y*, $\gamma_{s}$ is a State fixed effect, $\delta_{s}$ identifies the State trend *tr*, $\mu_{m}$ are month dummies, ${\mathrm{Pr}\_F\_age}_{smy}$ is the proportion of females by 5-year age group to control for age differences in the female population over time and across States, ${VIC}_{s}$ and ${NSW}_{s}$ are binary variables that identify if the birth rate is respectively for Victoria and New South Wales and ${Oth\_St}_{s}$ is a dummy for the other States/territories. ${First\_COVID}_{my}$ is a dummy for the months affected by COVID-19 policy measures until the start of the vaccine program (December 2020–November 2021) and ${Second\_COVID}_{my}$ is a dummy variable for the months in which vaccinations were progressively rolled-out. $\beta_{A;1}$ is the estimate of the percentage deviation of the birth rate in Victoria in the first period impacted by COVID-19 measures relative to the Victorian secular trend and $\beta_{A;2}$ and $\beta_{A;3}$ are respectively the estimates of the average percentage deviation for New South Wales and the other States/Territories relative to their trends. For the second year of the pandemic, $\beta_{B;1}$ estimates the average percentage deviation of the birth rate in Victoria and $\beta_{B;2}$ and $\beta_{B;3}$ the average deviation for New South Wales and for the other States/Territories.

Specification 2 involves splitting the months affected by COVID-19 into four sub-periods. The first period is December 2020 to March 2021 and corresponds to a period, 9 months earlier, in which babies were conceived under relatively similar COVID-19 restrictions in the different Australian States and Territories. The second period starts in April 2021 and corresponds to the commencement of the lockdown in Victoria in July 2020 and the few months preceding the vaccination program. The third period begins in December 2021 and coincides with the start of the COVID-19 vaccine campaign and a period free from prolonged lockdowns. The fourth period starts in March 2022 and corresponds to the commencement of the lockdown in New South Wales followed by Victoria one month later. The estimated equation is:

$$\log\left({BR}_{smy}\right)=\alpha +\gamma_{s}+\delta_{s}tr+\mu_{m}+\sum_{age=1}^{6}\theta_{age}{\mathrm{Pr}\_F\_age}_{smy}$$

$$+\left(\lambda_{A;1}{VIC}_{s}+\lambda_{A;2}{NSW}_{s}+\lambda_{A;3}{Oth\_St}_{s}\right)*{Same\_Meas\_First}_{my}$$

$$+\left(\lambda_{B;1}{VIC}_{s}+\lambda_{B;2}{NSW}_{s}+\lambda_{B;3}{Oth\_St}_{s}\right)*{Lock\_VIC}_{my}$$

$$+\left(\lambda_{C;1}{VIC}_{s}+\lambda_{C;2}{NSW}_{s}+\lambda_{C;3}{Oth\_St}_{s}\right)*{Same\_Meas\_Second}_{my}$$

$$+\left(\lambda_{D;1}{VIC}_{s}+\lambda_{D;2}{NSW}_{s}+\lambda_{D;3}{Oth\_St}_{s}\right)*{Lock\_VIC\_NSW}_{my}+\varepsilon_{smy}$$

where ${Same\_Meas\_First}_{my}$ is a dummy for the period between December 2020 and March 2021, ${Lock\_VIC}_{my}$ is a dummy for the period between April 2021 and November 2021, ${Same\_Meas\_Second}_{my}$ is a dummy for the months December 2021 to February 2022 and ${Lock\_VIC\_NSW}_{my}$ is a dummy that corresponds to the months March 2022 to June 2022. $\lambda_{A}$ s, $\lambda_{B}$ s, $\lambda_{C}$ s, and $\lambda_{D}$ s are estimates of the percentage deviations from the trends for Victoria, New South Wales, and the other States respectively in the first, second, third and fourth period.

Like specification 1, the third specification provides estimates of the average change in birth rates during a period when fertility decisions could have been influenced by the COVID-19 pandemic. Specification 3, however, exploits variation across SA2 areas, which requires using annual data on date of birth registration. The estimated equation is:

$$\log\left({BR}_{ay}\right)=\alpha +\gamma_{a}+\delta_{s}tr+\sum_{age=1}^{6}\theta_{age}{\mathrm{Pr}\_F\_age}_{ay}$$

$$+\left(\pi_{A;1}{VIC}_{a}+\pi_{A;2}{NSW}_{a}+\pi_{A;3}{Oth\_St}_{a}\right)*I_{2021}$$

$$+\left(\pi_{B;1}{VIC}_{a}+\pi_{B;2}{NSW}_{a}+\pi_{B;3}{Oth\_St}_{a}\right)*I_{2022}+\varepsilon_{ay}$$

where ${BR}_{asy}$ are birth rates of SA2 area *a* located in State *s* in year *y*, $\gamma_{a}$ is the SA2 area fixed effect, $\delta_{s}$ are the coefficients of State trends, ${\mathrm{Pr}\_F\_age}_{ay}$ is the proportion of females by 5-year age group in local area *a*, and $I_{2021}$ and $I_{2022}$ are indicators for respectively the year 2021 and the year 2022.

We then use the annual data on date of birth registration to estimate multiple equations that test if deviations from the trend in birth rates vary with the socio-economic characteristics of the area, such as geography, income, unemployment, proportion of residents whose first language is not English, and proportion of Indigenous people in the community. The socio-economic characteristics for each area are recovered from the 2021 Australian Census. In each of the five specifications the 2282 SA2 areas are split into two groups depending on whether:

i) the area belongs to the metropolitan area of a capital city or is a regional location;
ii) more than 20% of households have weekly equivalent incomes of less than AU$500^3^;
iii) the unemployment rate among people aged 15 to 64 is greater than 5%;
iv) English is not the first language at home for more than 20% of residents; and
v) more than 5% of the population are Indigenous.

In all these specifications we include two separate trends for each State/Territory – one for the local areas which have low values of the socio-economic characteristic and one for areas having high values (16 trends in total) – and estimate equations as follows:

$$\log\left({BR}_{ay}\right)=\alpha +\gamma_{a}+\delta_{gr;s}tr+\sum_{age=1}^{6}\theta_{age}{\mathrm{Pr}\_F\_age}_{ay}$$

$$+\left(\eta_{A;1}{VIC}_{a}+\eta_{A;2}{NSW}_{a}+\eta_{A;3}{Oth\_St}_{a}\right)*{Geo}_{a}*I_{2021}*{Group}_{a}+\left(\eta_{B;1}{VIC}_{a}+\eta_{B;2}{NSW}_{a}+\eta_{B;3}{Oth\_St}_{a}\right)*{Geo}_{a}*I_{2022}*{Group}_{a}+\varepsilon_{ay}$$

where ${BR}_{ay}$ are birth rates of the SA2 area *a* in year *y*, $\gamma_{a}$ is the SA2 area fixed effect, ${VIC}_{a}$ indicates if the area is in Victoria, ${NSW}_{a}$ if the area is in New South Wales and ${Oth\_St}_{a}$ if the area is in another State/Territory, ${Geo}_{a}$ indicates whether the area is a metropolitan or regional location, and ${Group}_{a}$ indicates if the area: is low- or high-income (in specification ii); has high- or low-unemployment (in iii); has a high or low proportion of non-English speaking residents (in iv); or has a high or low proportion of Indigenous people (in v). $\delta_{gr;s}$ *s* are the coefficients of the State-characteristic trends, and $\eta_{A}$ s and $\eta_{B}$ s are estimates of the different percentage deviations from trend respectively in 2021 and in 2022 after allowing for interactions between State, geography, and group of interest.

## 3 Results

### 3.1 Trends in birth rates

We start by investigating the long-term patterns of birth rates in Australia pre- and post-pandemic, covering the period 2011–2022. Fig. 1a and 1b plot birth rate indices calculated respectively from monthly data on date of birth and annual data on date of birth registration. The indices are constructed by dividing birth rates by the birth rate in October 2020 for monthly data and the rate in 2020 for annual data. By plotting indices instead of birth rates, these figures depict changes in birth rates relative to the last unaffected period.

**Fig. 1a:**
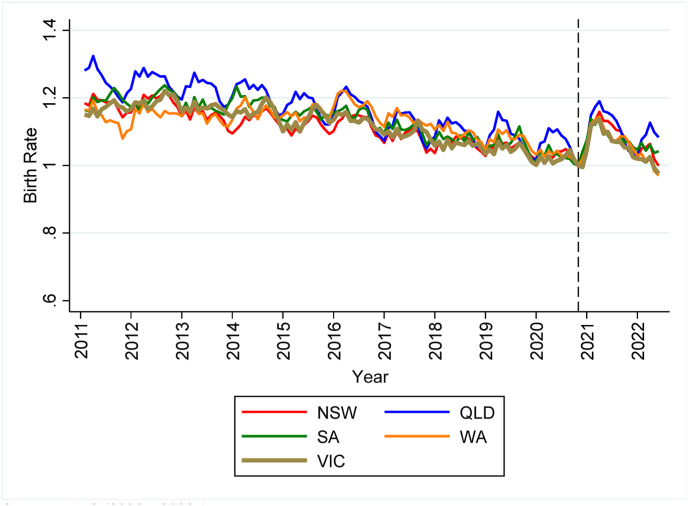
Birth rate indices (October 2020 = 1.0; monthly date of birth data).

*Sources*: ABS (2023a, 2023c).

**Fig. 1b:**
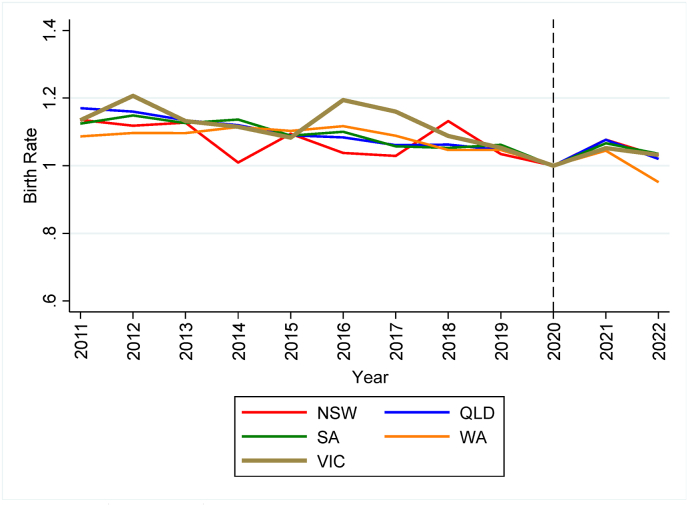
Birth rate indices (2020 = 1.0; annual date of birth registration data).

*Sources*: ABS (2023a, 2023c).

Both figures show declining trends in the years prior to the COVID-19 pandemic and a sharp, but short-lived, increase in fertility common to all States in the period affected by COVID-19 measures. Specifically, Fig. 1a reveals that the increase in birth rates starts in December 2020, nine months after the introduction of COVID-19 measures, and peaks between April and June 2021. In the months that follow, birth rates progressively return to the values of the predicted trends. The annual data depicted in Fig. 1b provides clear evidence of a surge in birth rates in 2021, followed by a subsequent decline in 2022, with rates remaining above the predicted trends.

To quantify the rate of growth in birth rates observed in Fig. 1a and 1b, Table 1 compares actual and predicted birth rates in the period affected by COVID-19 measures. Predicted birth rates are estimated by regressing birth rates on a linear trend and month dummies separately for each State using data preceding the COVID-19 pandemic. In Panel A, we use the monthly date of birth data and present actual and predicted rates for the months between December 2020 and November 2021 in the top half of Table 1. The bottom half of the table presents the rates for the period between December 2021 and June 2022. In Panel B, we use annual data on date of birth registration and calculate predicted rates for 2021 in the top half and for 2022 in the bottom half. For the first year of the pandemic, the results reported in both Panels A and B show large positive deviations from the predicted trend in all States, indicating more babies being born. However, Victoria, the State that experienced the longest lockdown in 2020, recorded one of the lowest rates of growth. Looking at Panel A, the birth rate in Victoria exceeded the pre-pandemic trend by 5.7% compared to larger positive deviations in most of the other States/Territories. A similar story can be inferred from Panel B. While the birth rate increased by between 2.0% and 8.0% in the other States/Territories, the rate in Victoria rose by just 1.0% relative to the trend.

**Table 1:** Actual, predicted, and deviations from pre-pandemic trends in birth rates.

State/Territory	Panel A: Date of birth (monthly data)			Panel B: Date of registration of birth (annual data)		
	Actual birth rates: Dec 2020 to Nov 2021	Predicted birth rates: Dec 2020 to Nov 2021	% deviation from predicted birth rates	Actual birth rates: 2021	Predicted birth rates: 2021	% deviation from predicted birth rates
VIC	56.8	53.7	5.7	55.9	55.4	1.0
NSW	61.2	56.5	8.5	63.2	59.4	6.4
QLD	59.0	55.0	7.3	61.8	57.3	8.0
WA	61.0	56.2	8.5	62.1	58.8	5.5
SA	56.6	53.6	5.7	58.3	55.3	5.3
TAS	56.9	53.9	5.5	58.3	55.4	5.3
ACT	52.8	50.3	4.9	53.2	51.9	2.4
NT	65.8	62.4	5.4	65.3	64.1	2.0
Australia	59.1	55.6	6.3	60.3	57.7	4.5
	Actual birth rates: Dec 2021 to June 2022	Predicted birth rates: Dec 2021 to June 2022	% deviation from predicted birth rates	Actual birth rates: 2022	Predicted birth rates: 2022	% deviation from predicted birth rates
VIC	31.2	30.5	2.0	54.9	54.6	0.4
NSW	33.6	32.2	4.1	60.3	58.7	2.7
QLD	33.2	31.7	4.9	58.6	56.3	4.1
WA	33.2	31.8	4.4	56.5	57.5	−1.8
SA	32.0	30.6	4.4	56.6	54.6	3.7
TAS	32.0	30.7	4.3	52.7	54.1	−2.6
ACT	30.2	28.3	6.4	51.7	50.4	2.7
NT	36.0	37.0	−2.8	61.4	63.0	−2.5
Australia	32.6	31.8	2.6	57.6	56.9	1.1

During the second phase of the pandemic, our results show a gradual return of the birth rates to pre-pandemic trends. Importantly, there are no significant differences between the two States that experienced prolonged lockdowns in 2021 and the other States. The bottom half of Panel A shows that Victoria and New South Wales surpassed the pre-pandemic trend by 2.0% and 4.1%, respectively. This contrasts with deviations in the range of −2.8% to 6.4% observed in the other States and Territories. Results in the lower half of Panel B show that the increase relative to the trend in 2022 in Victoria and New South Wales was 0.4% and 2.7%, respectively. This is in comparison to a mix of negative and positive deviations observed in the other States. Overall, these findings, confirmed by data on total fertility rates reported in Appendix Table A1,^4^ suggest a temporary baby boom associated with the COVID-19 pandemic, albeit with considerable variations across the different States.

### 3.2 Changes in birth rates under COVID-19 restrictions

Table 2 presents estimates of birth rate deviations during the period affected by COVID-19 restrictions, derived from the OLS regression analysis. The first two rows present estimates for specifications 1 and 2, both of which use monthly date of birth data. The estimates for specification 1 show that birth rates observed between December 2020 and November 2021 exceeded pre-pandemic trends. Birth rates in Victoria, however, increased by 6.2% relative to the pre-pandemic trend. This is significantly less than the 8.6% in New South Wales and the average 7.9% growth experienced by the other States/Territories.

**Table 2:** Deviations from State trends in the period affected by COVID-19 measures.

	Victoria	New South Wales	Rest of Australia	Test of equality
*Specification 1 (Monthly date of birth data)*				
Lockdowns and restrictions prior to vaccine roll-out	*β*_A;1_	*β*_A;2_	*β*_A;3_	*β*_A;1_ = *β*_A;2_
	.062*** [0.045, 0.080]	.086*** [0.068, 0.104]	.079*** [0.068, 0.090]	144.49*** β_A;1_ = *β*_A;3_ 7.31**
Lockdowns and restrictions during vaccine roll-out	*β*_B;1_ .023 [-0.008, 0.055]	*β*_B;2_ .043** [0.013, 0.074]	*β*_B;3_ .061*** [0.045, 0.078]	*β*_B;1_ = *β*_B;3_ 19.07*** β_B;2_ = *β*_B;3_ 6.11**
*Specification 2 (Monthly date of birth data)*				
Partial national lockdown and restrictions	*λ*_A;1_	*λ*_A;2_	*λ*_A;3_	*λ*_A;1_ = *λ*_A;2_
	.067*** [0.051, 0.084]	.077*** [0.060, 0.094]	.077*** [0.063, 0.091]	29.43*** λ_A;1_ = *λ*_A;3_ 2.82
Lockdown in Victoria	*λ*_B;1_	*λ*_B;2_	*λ*_B;3_	*λ*_B;1_ = *λ*_B;2_
	.061*** [0.042, 0.080]	.093*** [0.073, 0.112]	.081*** [0.069, 0.093]	183.76*** λ_B;1_ = *λ*_B;3_ 6.17**
Start vaccine roll-out and minor restrictions in place	*λ*_C;1_ .049*** [0.025, 0.073]	*λ*_C;2_ .053*** [0.030, 0.075]	*λ*_C;3_ .063*** [0.026, 0.099]	*λ*_C;1_ = *λ*_C;2_ 1.20 *λ*_C;1_ = *λ*_C;3_ 0.97
Lockdowns in Victoria and New South Wales	*λ*_D;1_ .003 [-0.036, 0.043]	*λ*_D;2_ .038* [-0.001, 0.076]	*λ*_D;3_ .062*** [0.038, 0.086]	*λ*_D;1_ = *λ*_D;3_ 9.67** λ_D;2_ = *λ*_D;3_ 1.93
*Specification 3 (annual date of registration of birth data)*				
Lockdowns and restrictions in 2021	*π*_A1_ .026*** [0.014, 0.039]	*π*_A2_ .085*** [0.073, 0.096]	*π*_A2_ .065*** [0.057, 0.073]	*π*_A1_ = *π*_A2_ 53.96*** π_A1_ = *π*_A3_ 28.47***
Lockdowns and restrictions in 2022	*π*_B1_ .026*** [0.011, 0.041]	*π*_B2_ .059*** [0.046, 0.072]	*π*_B3_ .021 [0.009, 0.031]	*π*_B1_ = *π*_B3_ 0.41 *π*_B2_ = *π*_B3_ 24.40***

*Notes*: Figures in parentheses are 95% confidence intervals. In specifications 1 and 2, standard errors are clustered at State level. In specification 3, standard errors are clustered at small geographic area (SA2) level. ***p < 0.01, **p < 0.05, *p < 0.1.

During the second phase of the pandemic, which began in February 2021, birth rates continued to exceed pre-pandemic trends, albeit to a lesser extent than in the first year. The rise in birth rates in the two States subjected to an almost four-month lockdown was less pronounced compared to the average of the other States/Territories, as confirmed by the Wald-tests for equality of coefficients reported in Table 2.

Specification 2 allows deviations to vary between the months in which similar restrictions were enforced across all States and the months in which Victoria and New South Wales experienced the lockdown. In the first year of the pandemic, Victoria experienced lower growth in birth rates during lockdown, with positive deviations remaining stable at around 6%. A similar pattern can be inferred in the second phase of the pandemic. Although birth rates were gradually reverting to pre-pandemic trends, in the months with relatively similar restrictions, positive deviations from the trend were much lower in the months in which Victoria and New South Wales experienced a lockdown. Notably, in Victoria the deviation from the trend between March and June 2022 was not statistically different from zero.

Finally, estimates of specification 3 show that results obtained when using data on the dates births were registered are consistent with those using monthly data on the actual date of birth. Estimates reported in the last row of Table 2 show that the rise in birth rates in the first year of the pandemic was more substantial in both New South Wales and the other States than in Victoria. Our results for 2022 reveal larger positive deviations in New South Wales compared to Victoria and the other States/Territories. This could suggest a potentially faster recovery in birth rates in New South Wales following the end of the lockdown.

### 3.3 Changes in birth rates by socio-economic characteristics of the area

In Fig. 2a, Fig. 2b, Fig. 3a, Fig. 3b, we present estimates of changes in birth rates by socio-economic characteristics of areas. Fig. 2a, Fig. 3a correspond to deviations in 2021 while Fig. 2b, Fig. 3b pertain to 2022. Fig. 2aa and 2b shows a surge in birth rates across all regions compared to the State-trend. This rise in birth rates was relatively similar in both regional and metropolitan areas during the two years of the COVID-19 pandemic. This pattern is found both in Victoria and in the other States. In New South Wales, in 2022, regional areas exhibited larger positive deviations than Greater Sydney possibly due to shorter lockdowns experienced in some regional locations.

**Fig. 2a:**
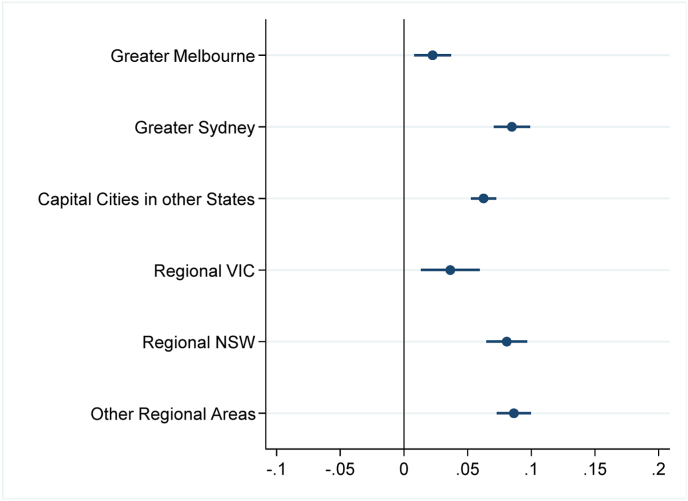
Birth rate deviations in 2021 from State-geography trends *Note:* The figure depicts coefficient estimates and 95% confidence intervals. Standard errors clustered at small geographic area (SA2) level.

*Source*s: ABS (2023a, 2023c).

**Fig. 2b:**
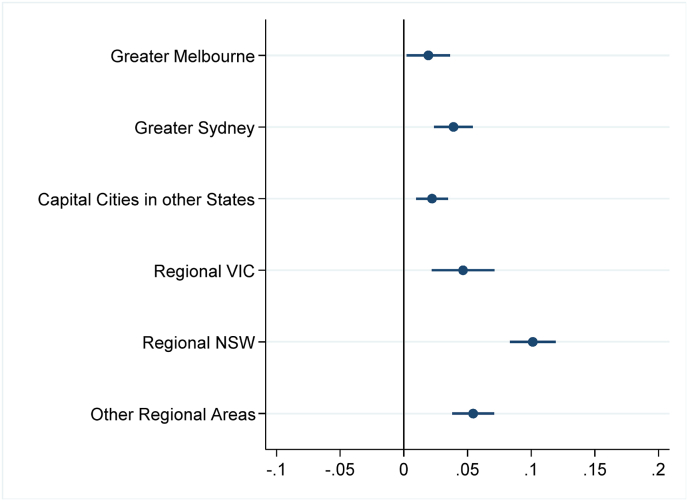
Birth rate deviations in 2022 from State-geography trends *Note*: The figure depicts coefficient estimates and 95% confidence intervals. Standard errors clustered at small geographic area (SA2) level.

*Source*s: ABS (2023a, 2023c).

**Fig. 3a:**
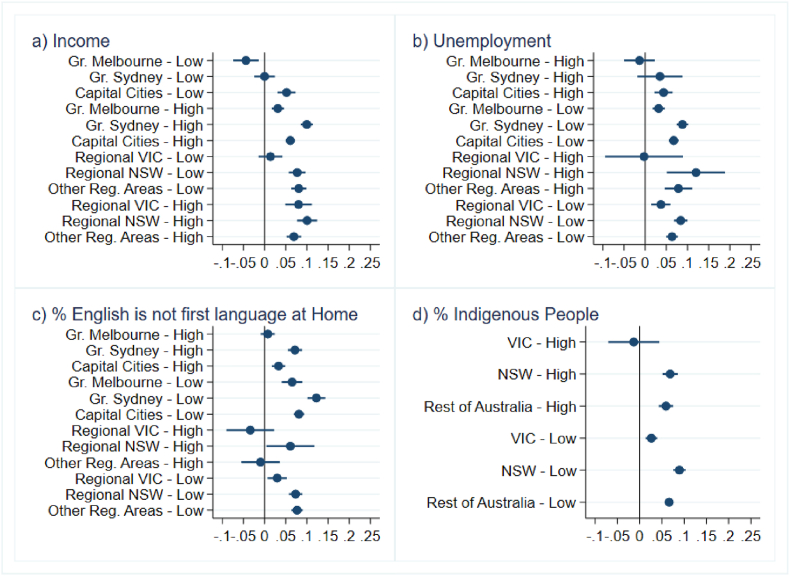
Birth rate deviations in 2021 from State-characteristic trends, by area socio-economic characteristics *Note:* Figures depict coefficient estimates and 95% confidence intervals. Standard errors clustered at small geographic area (SA2) level.

*Sources*: ABS (2023a, 2023c).

**Fig. 3b:**
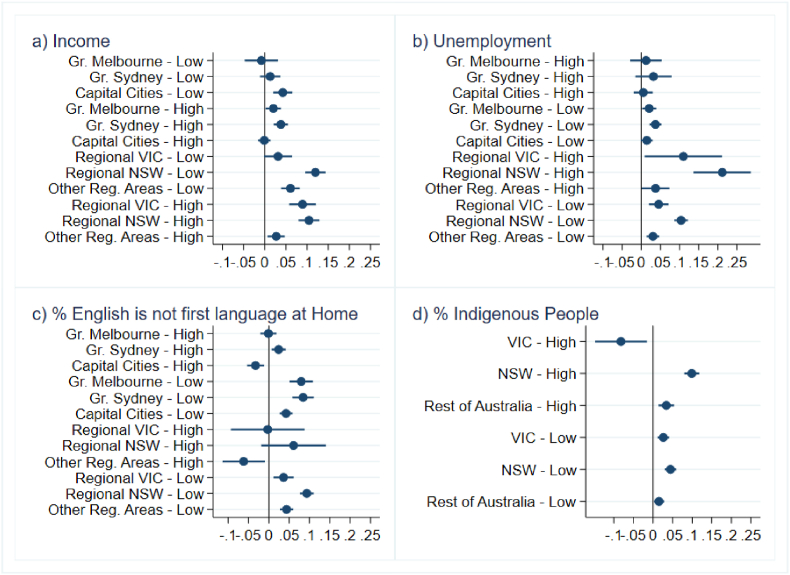
Birth rate deviations in 2022 from State-characteristic trends, by area socio-economic characteristics *Note*: Figures depict coefficient estimates and 95% confidence intervals. Standard errors clustered at small geographic area (SA2) level.

*Sources*: ABS (2023a, 2023c).

Panels A and B of Fig. 3a show that in 2021, birth rates either increased less or even declined in areas with higher proportions of low-income residents and regions with elevated unemployment rates, and this was particularly noticeable in low-income or high-unemployment areas in Melbourne. Conversely, fertility increased considerably in high-income or low-unemployment areas. Patterns in regional areas suggest smaller increases in low-income or high-unemployment areas. However, confidence intervals overlap in most cases, suggesting that the differences between high-income and low-income areas, and between low-unemployment and high-unemployment areas, are not statistically significant. Despite this, we find higher growth in birth rates in high-income regional locations compared to low-income areas in Victoria.

Panel C of Fig. 3a reveals smaller increases in birth rates in areas with more language diversity, notably in Melbourne and regional Victoria. Birth rates in areas with higher language diversity in Sydney and other States also rose but to a lesser extent than in less diverse areas. Panel D indicates moderate or no significant deviations in birth rates in Victoria based on the proportion of Indigenous people, while other States/Territories show positive deviations from trends, regardless of the Indigenous population's proportion in the area.

Fig. 3b reveals a shift in the association between birth rate changes and socio-demographic characteristics in 2022. Panels A and B show reduced rates of growth in birth rates in high-income or low-unemployment metropolitan areas compared to 2021, a pattern that is consistent across all States. Notably, the increase in birth rates remained sluggish in language-diverse areas in the second pandemic year, a trend that is particularly evident in metropolitan regions (Panel C). While significant differences exist in metropolitan areas, regional estimates are less precise due to the limited number of rural language-diverse locations.

Overall, these findings highlight the continued influence of socioeconomic and cultural factors on fertility patterns.

## 4 Discussion

We explored changes in birth rates in Australia during the COVID-19 pandemic using natality data from 2011 to 2022 at both State and small regional area levels. We investigated the influence of lockdowns on birth rates and variations across regions characterized by diverse factors, including Indigenous population, unemployment rates, low-income, and non-English speaking residents.

Our initial findings revealed an immediate increase in birth rates in the first year of the pandemic, aligning with trends in the US (Bailey et al., 2023) and various North European countries (e.g., Sobotka et al., 2023; Nisén et al., 2022; Lappegård, Kristensen, Dommermuth, Minello, & Vignoli, 2022). This surge can be attributed to low infection rates due to border closures and the government's proactive COVID-19 approach, alongside extensive income support programs that mitigated economic strains (Breunig & Sainsbury, 2023). Additionally, unplanned pregnancies due to diminished access to contraception and abortion during 2020 may have also contributed to the observed rise in fertility.

In Australia, the rise in fertility in 2021 was driven mainly by individuals in high-income areas. This is consistent with higher fertility rates among college-educated women reported in other countries (Bailey et al., 2023; Lappegård et al., 2022), supporting the notion that affluent young couples had the opportunity to reassess their family plans while spending more time together and slowing down their busy life.

Victoria, however, experienced significantly lower birth rate growth, likely driven by the imposed lockdown. Specifically, following a state of emergency declaration in July 2020, Victoria's government periodically revised stay-at-home orders without giving a clear end date to the lockdown, which may have led couples, especially in low-income areas, to postpone conceiving. The usual culprit is the uncertainty surrounding job prospects, which has been found to influence individual decision-making around family formation (e.g., Autor et al., 2019; Chandra et al., 2018; Schaller, 2016). However, due to the aggregate nature of our data, we were unable to investigate specific reasons guiding fertility decisions at the individual level, highlighting an avenue for future research.

Finally, examining data from 2022, and in line with the findings of Sobotka et al. (2023), we found a short-lived surge in birth rates, peaking 6–8 months after the global pandemic declaration and gradually returning to pre-pandemic levels. In the second year, marked by new lockdowns, States with both moderate and severe restrictions showed similar rates of growth in birth rates. The decline in birth rate growth among residents in high-income metropolitan locations and the continued fertility decline in language-diverse areas were notable contributors to the return to pre-pandemic trends. The latter is almost certainly a direct result of the way Australia managed migration and international borders. The ban on non-citizens and non-residents entering Australia (commencing March 20, 2020 and lasting until 21 February 2022 for vaccinated persons) led to a significant reduction in immigration: from a net overseas migration inflow of 247,600 (around 1% of the Australian population) in 2019 to a net outflow of 5000 in 2020 and a net inflow of 6800 in 2021 (ABS 2023b).^5^ Given that birth rates are higher among females born overseas (see Table 3) (see also Baffour et al., 2022), it is very likely that the restrictions on migration in 2020 and 2021 negatively impacted fertility rates, particularly in areas where new immigrants tend to settle.

**Table 3:** Birth rates by mother's country of birth.

Mother's country of birth	NSW	VIC	QLD	SA	WA	TAS	NT	ACT	Total
Australia	57.9	53.6	55.9	55.4	57.7	54.6	55.7	46.3	55.8
Overseas	61.5	61.9	59.1	59.5	64.4	58.3	67.9	58.8	61.4
Overseas excluding NZ, UK, Ireland, USA, Canada	60.8	62.1	57.6	59.9	61.8	58.8	66.8	59.2	60.8

*Notes:* Birth rates calculated from ABS Census data using births of the year prior Census night (10^th^ August 2021).

## 5 Conclusion

Our study documented the dynamic changes in birth rates across Australia post-pandemic. During the first year, birth rates increased across all Australian States, including Victoria, despite its strict lockdown measures. The areas showing slower growth in birth rates were those with higher unemployment, lower incomes, and a larger percentage of non-English speaking residents. Moving into the second year of the pandemic, birth rates gradually returned to their pre-pandemic trend, at least in the major (i.e., capital) cities. Overall, we are drawn to the conclusion that the pandemic was associated with a small upward bump in birth rates in Australia that was temporary, and which was less marked in Victoria following the 2020 lockdown, but with little or no evidence that the subsequent lockdowns in 2021 in both Victoria and New South Wales had any effect.

## Ethical statement

Ethical approval for the study was not required as the study does not use data collected from human subjects.

## CRediT authorship contribution statement

**Irma Mooi-Reci:** Writing – review & editing, Writing – original draft, Supervision, Project administration, Investigation, Funding acquisition, Conceptualization, Prof dr. **Mark Wooden:** Writing – review & editing, Supervision, Funding acquisition, Conceptualization. **Federico Zilio:** Writing – review & editing, Visualization, Validation, Methodology, Formal analysis, Data curation.

## Declaration of competing interest

None.

## Figures and Tables

**Table A.1:** Actual, predicted, and deviations from pre-pandemic trends in Total Fertility Rates (TFR)

State/Territory	Actual TFRs: 2021	Predicted TFRs: 2021	% deviation from predicted TFRs	Actual TFRs: 2022	Predicted TFRs: 2022	% deviation from predicted TFRs
VIC	1.53	1.50	1.9	1.51	1.47	3.1
NSW	1.77	1.65	7.7	1.71	1.62	5.7
QLD	1.79	1.64	9.6	1.71	1.60	6.9
WA	1.76	1.64	6.9	1.62	1.60	0.8
SA	1.66	1.56	6.7	1.62	1.52	6.3
TAS	1.64	1.56	4.8	1.49	1.51	−1.1
ACT	1.45	1.41	3.1	1.41	1.36	4.1
NT	1.83	1.76	3.6	1.73	1.72	0.1
Australia	1.70	1.60	6.1	1.63	1.57	4.2

## Data Availability

Data will be made available on request.

## Footnotes

3 The median household equivalent weekly income for the Australian population was $959 in the 2019/20 financial year (ABS 2022a).

## Footnotes

1 See also Ghaznavi et al. (2022) for Japan, Lima et al. (2022) and Moreno-Louzada and Menezes-Filho (2023) for Brazil, Nisén et al. (2022) for Finland, and Pesando and Abufhele (2023) for Chile.

2 To compare statistics for small regional areas in Australia, the ABS has designed 2450 SA2 areas that aim to represent communities in which residents interact socially and economically. The population of SA2 areas range between 3000 and 25,000.

4 The total fertility rate is a measure of the average number of children that a woman is expected to give birth to over her lifetime. Unlike the birth rate, it adjusts for the age structure of the population. In our analysis we draw on birth rates as data on births by mother's age are unavailable at the local area level and by month of birth. In sections 3, 3.2.3 we deal with this issue by controlling for the distribution of women across 5-year age groups within each local area.

5 Derived from net overseas migration numbers reported in a table attached to ABS (2022b). Considering only the period of international border closure (from second quarter 2020 to third quarter 2021), overseas migration recorded a net outflow of 107,100 (ABS 2023b).
